# Multimodal Neuroimaging in Rett Syndrome With *MECP2* Mutation

**DOI:** 10.3389/fneur.2022.838206

**Published:** 2022-02-23

**Authors:** Yu Kong, Qiu-bo Li, Zhao-hong Yuan, Xiu-fang Jiang, Gu-qing Zhang, Nan Cheng, Na Dang

**Affiliations:** ^1^Department of Medical Imaging, Affiliated Hospital of Jining Medical University, Jining, China; ^2^Department of Pediatrics, Affiliated Hospital of Jining Medical University, Jining, China; ^3^Department of Pediatric Rehabilitation, Affiliated Hospital of Jining Medical University, Jining, China

**Keywords:** Rett syndrome, multimodal neuroimaging, magnetic resonance imaging, positron emission tomography, *MECP2*

## Abstract

Rett syndrome (RTT) is a rare neurodevelopmental disorder characterized by severe cognitive, social, and physical impairments resulting from *de novo* mutations in the X-chromosomal methyl-CpG binding protein gene 2 (*MECP2*). While there is still no cure for RTT, exploring up-to date neurofunctional diagnostic markers, discovering new potential therapeutic targets, and searching for novel drug efficacy evaluation indicators are fundamental. Multiple neuroimaging studies on brain structure and function have been carried out in RTT-linked gene mutation carriers to unravel disease-specific imaging features and explore genotype-phenotype associations. Here, we reviewed the neuroimaging literature on this disorder. MRI morphologic studies have shown global atrophy of gray matter (GM) and white matter (WM) and regional variations in brain maturation. Diffusion tensor imaging (DTI) studies have demonstrated reduced fractional anisotropy (FA) in left peripheral WM areas, left major WM tracts, and cingulum bilaterally, and WM microstructural/network topology changes have been further found to be correlated with behavioral abnormalities in RTT. Cerebral blood perfusion imaging studies using single-photon emission CT (SPECT) or PET have evidenced a decreased global cerebral blood flow (CBF), particularly in prefrontal and temporoparietal areas, while magnetic resonance spectroscopy (MRS) and PET studies have contributed to unraveling metabolic alterations in patients with RTT. The results obtained from the available reports confirm that multimodal neuroimaging can provide new insights into a complex interplay between genes, neurotransmitter pathway abnormalities, disease-related behaviors, and clinical severity. However, common limitations related to the available studies include small sample sizes and hypothesis-based and region-specific approaches. We, therefore, conclude that this field is still in its early development phase and that multimodal/multisequence studies with improved post-processing technologies as well as combined PET–MRI approaches are urgently needed to further explore RTT brain alterations.

## Introduction

The postnatal stage is a significantly dynamic brain growth and development period characterized by both macrostructural and microstructural changes. Macrostructural changes include global/regional brain volume and weight modifications as well as changes in the regional gray matter (GM)/white matter (WM) ratio, and microstructural changes mainly involve cell differentiation, myelination, synaptogenesis, and cortical layering (1, 2). As a severe neurological disease, Rett syndrome (RTT) leads to neurodevelopmental abnormalities in this crucial period, primarily arising from *de novo* mutations in the X-chromosomal methyl-CpG binding protein gene 2 (*MECP2*) (3–5). RTT is manifested as a severe cognitive, social, and physical impairment. Neuroimaging has been used extensively to assess the brain structure, connectivity, and function, providing a valuable tool to link neuronal activity, anatomic structure, cerebral function, and several complex clinical events (6). The application of neuroimaging to RTT has been explored for decades. Various imaging techniques have been used to answer the fundamental questions about the biological basis of RTT and to characterize *in vivo* disease pathology. Neuroimaging studies on RTT provide essential insights into anatomical, functional, metabolic, and dynamic changes in the brain reflecting the biological effects of *MECP2* mutations and allow to monitor future therapeutic outcomes. The present article reviews the current applications of multimodal neuroimaging to RTT and provides potential future directions in this field.

## Methods

A formal literature review was conducted on PubMed (https://pubmed.ncbi.nlm.nih.gov/) with the following search terms: (“Rett syndrome” OR “*MECP2*”) AND (“magnetic resonance imaging” OR “positron emission tomography” OR “single-photon emission tomography” OR “diffusion MRI” OR “diffusion tensor imaging” OR “diffusion kurtosis imaging” OR “neurite orientation dispersion and density imaging” OR “magnetic resonance spectroscopy” OR “cerebral blood flow” OR “arterial spin labeling” OR “MRI” OR “PET” OR “SPECT” OR “MRS” OR “DTI” OR “DKI” OR “NODDI” OR “ASL”). All searches were updated in October 2021. The retrieved results were then filtered according to the inclusion criteria reported as follows.

The study inclusion criteria required articles of full-text publications in English or translated into English. The research subjects of eligible articles were RTT subjects with *MECP2* mutations. We set the primary focus of our review on the studies applying multimodal neuroimaging to RTT with *MECP2* mutations, so both human and animal studies were included. Human studies are summarized in Supplementary Table S1, and animal studies are summarized in Supplementary Table S2.

Case reports and conference communications were excluded. Also, non-English articles and studies on *MECP2* duplication syndrome or RTT caused by the mutations of genes other than *MECP2* were not considered.

## RTT With *MECP2* Mutation

Rett syndrome is a severe neurodevelopmental disorder named after the Austrian pediatrician Andreas Rett, who first described this disorder in 1966 (7). RTT almost exclusively affects women, and ~1 in 10,000–15,000 girls have this disease (8). In RTT, normal development is usually observed up to 7–18 months, followed by the developmental regression in which previously acquired skills are lost (4, 9). Most patients progress through four clinical stages and show various clinical features. The diagnostic criteria and clinical stages for RTT are presented in Table 1 (10–13).

**Table 1:** Diagnostic criteria and clinical stages for Rett syndrome (RTT).

**Main criteria**	**Supportive criteria**
**Diagnostic criteria**	
1. Regression followed by recovery or stabilization	1. Respiratory disturbances
2. Partial or complete loss of acquired purposeful hand skills and spoken language	2. Bruxism when awake
3. Gait abnormalities	3. Sleep disruption
4. Stereotypic hand movements	4. Abnormal muscle tone
	5. Peripheral vasomotor disorders
	6. Scoliosis/kyphosis
	7. Growth delays
	8. Small and cold hands and feet
	9. Laughing/screaming
	10. Insensitive to pain
	11. Intense eye communication
**Exclusion criteria**	
1. Brain injury secondary to trauma, neurometabolic disease, or severe infection	
2. Grossly abnormal psychomotor development in first 6 months of life	
**Clinical stages**	
	**I Early onset stage**	**II** **Rapid destructive stage**	**III Pseudo-stationary stage**	**IV** **Late deterioration stage**
Onset time	Age 6–18 months	Age 1–4 years	Age 4–8 years	After Age 8 years
Duration	Months	Weeks to Months	Years to Decades	Decades
Characteristics	Developmental arrest, diminished interest in play, hand waving, and decelerating head growth	Developmental deterioration, severe dementia, loss of hand skills and spoken communication, irregular breathing, and appearance of seizures	Stabilization, gross motor dysfunction, gait apraxia, jerky truncal ataxia, frequent seizures	Decreasing mobility, loss of independent ambulation

*According to the revised diagnostic criteria and nomenclature of Rett Search Consortium (10); Clinical stages according to Hagberg and Witt-Engerstrom (11)*.

More than 90% of classical RTT arises from *de novo* mutations in *MECP2* (3–5). As a transcriptional regulator, *MECP2* is expressed widely throughout the whole body and particularly in the mature neurons of the brain (14, 15). It plays a crucial role in neuronal development, differentiation, and synaptic plasticity (16–18). It is becoming clear that *MECP2* is almost unexpressed in early embryonic stages in mice and humans, and its expression gradually increases in the postnatal stage and childhood (19, 20). Researchers have identified hundreds of different *MECP2* mutations contributing to distinct clinical phenotypes and disease severities (18, 21, 22). There has been a consensus in eight “hotspot” *MECP2* mutations in RTT (R106W, R133C, T158M, R168X, R255X, R270X, R294X, and R306C), which affect >60% of the documented cases (23). Extensive sample statistics found that patients with R133C, R294X, R306C, or T158M manifest with milder phenotypes, while the cases with R106W, R168X, R255X, or R270X, or large deletions show more severe disease forms (24, 25). In addition, several studies on *MECP2* deletions have found that specific neuronal populations are involved in distinct pathophysiological mechanisms leading to different clinical phenotypes (26–35). These details are described in Supplementary Table S3.

Although some of the disease mechanisms have been unraveled, genetic heterogeneity, genotype-phenotype interplays, and epigenetic factors in RTT are still not fully understood. A few previous studies have reported that a broad spectrum of disabilities in girls with RTT reflects the pervasive abnormalities of brain growth (especially the developmental phase of intense synaptogenesis) and connectivity (the formation of neural signaling pathways) (36, 37). However, the aforementioned findings were almost based on postmortem brain tissue analyses of patients with RTT, while *in vivo* monitoring of the underlying pathophysiological processes is more helpful to understand the development of RTT disease. Although there is still no cure for RTT and the available treatments are mainly symptomatic (Supplementary Table S3), alleviating symptoms, reducing pain and discomfort, and increasing the quality of life are essential for both patients and caregivers (38). Early identification is a prerequisite for the implementation of targeted and timely therapeutic approaches. A few studies have shown that early intervention can delay the developmental regression in girls with RTT, and the treatment of symptoms can alleviate the associated pain. Thus, searching for neurofunctional markers for early diagnosis, discovering potential targets for effective therapies, and exploring efficacy evaluation indicators are pivotal.

## Morphologic MRI in RTT With *MECP2* Mutation

The addition of MRI to the diagnostic armamentarium of neurodevelopmental disorders may be considered as a revolution in this field. Of all the three-dimensional imaging techniques, MRI has the best soft-tissue contrast. Moreover, different MRI sequences can reflect corresponding tissue contrasts, thus providing a wealth of information about the brain structure and tissue microstructure (39, 40). MRI has been identified as an effective morphological imaging method for detecting structural abnormalities in RTT due to its high resolution and its optimized GM/WM delineation (41).

Rett syndrome is characterized by acquired microcephaly (42). Accordingly, brain MRI studies on patients with RTT have shown global brain atrophy and specific regional GM/WM reductions in the frontal and temporal lobe, hippocampus, caudate nucleus, striatum, thalamus, midbrain, and WM tracts (43–45). Animal studies have been further conducted to explore the relationship between macroscopic brain structure changes and RTT phenotype. Saywell et al. investigated *MECP2*-null mice, a widely recognized experimental model of RTT, using conventional brain MRI and found a global reduction in its brain size, a feature constantly observed in patients with RTT (46). Reduced cerebellum size may account for some of the neurological signs observed in RTT, including cognition and motor coordination impairments. Moreover, the authors found significant thinning of some specific structures, such as the motor cortex and the corpus callosum (46). Elsewhere, Allemang-Grand et al. used a high-resolution MRI with deformation-based morphometric approaches to examine the brain structure of different mouse models carrying *MECP2* mutations, demonstrating the severity of the mutation and the stage of behavioral impairment were associated with the degree of neuroanatomical changes (47).

Although qualitative analysis is helpful for clinical decision-making, it does not provide quantitative values to monitor the developmental status (48). In this context, it is noteworthy that only a few quantitative studies on the brain morphology related to RTT have been carried out. In the field of animal studies, Patrick et al. created an MRI atlas for detailed cerebellar volume analysis and quantitatively investigated genetic effects on this structure (49). Using this approach, the authors were able to reveal a complex interplay between *MECP2* mutations, cerebellar volumetric changes, repetitive behaviors, and learning (49). In the field of human studies, Carter et al. used complementary semiautomated Talairach- and voxel-based approaches to study MRI scans acquired in female cases carrying *MECP2* mutations. The authors provided novel pieces of evidence on selective reductions of dorsal parietal GM and the preservation of the occipital cortex in RTT (50), and further reported a correlation between anterior frontal lobe reduction and clinical severity. As regards cortical WM, mild and diffuse reductions have been previously reported and linked to axonal pathology (50). Notably, studies in this field have selectively shown decreased volumes of the cerebrum, cerebellum, and caudate nucleus (43, 44, 50–53). Surface-based morphological approaches, including cortical gyrification and regional cortical thickness evaluation, have not been fully explored. Previous studies using quantitative analyses (including surface- and voxel-based measurements) observed no significant differences in global cortical gyrification, thickness, and volumes, as well as in regional cortical thickness between patients with RTT/Rett-like (RTT-l) (cases carrying the *MECP2* mutation but not fulfilling the diagnostic criteria for RTT) and normal controls (54), but evidenced a significant volumetric reduction of the cerebellum. Given that the patients with RTT/RTT-l included in this study were younger than those described in other reports, it is plausible to hypothesize that cerebellar volume reductions may precede regional cortical atrophy, providing a potential early diagnostic marker in patients with RTT/RTT-l (54).

In addition to morphological studies, MRI sequences can provide other information about brain changes in patients with RTT; for example, the underlying tissue microstructure can be examined using diffusion MRI (dMRI) (55–57), functional information can be gathered using functional MRI (fMRI) (57), metabolic differences can be identified using magnetic resonance spectroscopy (MRS) (46), and cerebral blood flow (CBF) can be assessed using arterial spin labeling (ASL) (58, 59).

## Diffusion MRI

Complex microstructural changes are increasingly recognized as significant contributors to neurodevelopmental disease even in the absence of gross brain morphologic changes (2). dMRI can noninvasively monitor microstructural changes and identify the implicated neural networks by mapping the distribution and movement of water molecules in the brain tissue. Recent studies have shown that it is also sensitive to cortical microstructure properties, such as radial and tangential fiber populations and neuropil volume fraction (60–65). Previous morphologic studies have suggested that the decreased cortical WM volume observed in RTT may be secondary to neuronal somata changes and primary axonal disturbances (6, 66). However, confirming this hypothesis requires the application of complementary imaging approaches such as dMRI. This technique also provides a new perspective for understanding the underlying pathological mechanism, assisting in diagnosis, and evaluating the neurobiological bases of the observed symptoms.

Diffusion tensor imaging (DTI) is an MRI technique based on the physical principle of water molecule diffusion restrictions across WM tracts (67). The tensor can derive several parameters, including fractional anisotropy (FA) and mean diffusivity (MD). FA represents the degree of anisotropy of the diffusion, which is a sensitive imaging biomarker for axonal organization and myelin integrity (2). MD represents the magnitude of diffusion, which is a commonly used scalar measurement.

In patients with RTT, a reduced FA has been identified in left peripheral WM areas (including middle temporal, middle occipital, precuneus, and postcentral regions), left major WM tracts (such as the superior longitudinal fasciculus, sagittal stratum, and corpus callosum), and the cingulum bilaterally (48). By studying the correlation between DTI-derived FA measurements and specific clinical features in patients with RTT, Mahmood et al. obtained the following findings: firstly, FA in the superior longitudinal fasciculus was significantly associated with speaking abilities; secondly, FA reductions in the anterior cingulate gyrus were associated with the characteristic mood and behavioral changes often observed in patients with RTT; and thirdly, the common observation of intact visual capabilities might be in accordance with the normal to increased FA values identified in the posterior corona radiate (68). Taken together, these preliminary studies suggest that DTI may represent a valuable noninvasive technique to assess WM tract pathological processes and add specificity to the assessment of RTT clinical severity.

Diffusion tensor imaging obtains the two types of information: quantitative water diffusion parameters described above and global brain WM organization metrics. The latter includes maps of fiber bundle orientation using color-coded DTI maps and a more precise delineation of specific fiber pathways by using tractography, which is based on identifying tracts using the color maps (67, 69). Fiber bundles are delineated using the software that traces the trajectory of the vectors representing water diffusion. Fiber delineation/reconstruction studies usually focus on specific pathways, which have been postulated to be involved in the disease. To date, tractography technology has been the most widely applied technology in animal studies. Wang et al. reported abnormal brain WM developmental dynamics and network topological organizations in RTT monkey models across different clinical stages *via* longitudinal DTI (70). They revealed that the *MECP2* mutation could lead to early protracted WM myelinization affecting later synaptic pruning and inducing abnormal functional segregation of the brain in RTT (70). Early abnormal WM development may be the underlying neural mechanism for some of the significant abnormal clinical neurobehavioral phenotypes, and it may also serve as an early predictor of RTT (70).

However, the major limitation of DTI quantitative parameters is that FA and MD provide nonspecific measures of pathology. The assumption of a single compartment with Gaussian diffusion does not adequately model the involved biological systems, like the WM, with its complex fiber architectures, or the GM, where diffusion is relatively isotropic, and this model fits poorly (71). A variety of more advanced models of tissue diffusion providing alternative parameters are now available, such as diffusion kurtosis imaging (DKI) or neurite orientation dispersion and density imaging (NODDI). These have the potential to identify previously unseen structural abnormalities and improve our understanding of underlying microstructural changes. However, no DKI or NODDI studies on RTT have been reported so far.

## Cerebral Blood Perfusion Imaging

The most widely used imaging methods of cerebral blood flow evaluation are single-photon emission CT (SPECT) or PET imaging (72–74), and the most consistently reported finding in this field is the presence of frontal hypoperfusion. Nielsen et al. studied seven patients with RTT with ^133^Xe SPECT scans and found that global CBF was decreased and the hypoperfusion foci were located mainly in prefrontal and temporoparietal areas (72). Burroni et al. performed ^99m^Tc-ECD brain SPECT imaging on 12 girls with classical RTT and a control group of normal children, also attaining similar findings (74). These observations align with the neuropathological evidence of a global reduction in brain size and an alteration in the dendritic and synaptic trees in RTT. Moreover, no significant right-to-left asymmetry was found in any regions of interest of the cortex, which confirms that RTT is a diffuse and nonfocal neurological disorder (74). Brain perfusion abnormalities were seen more often in stage IV patients with RTT rather than in stage III patients with RTT, supporting the notion that CBF reductions probably reflect clinical disease progression (74, 75). Researchers have also reported a reduced CBF in patients with normal brain MRI scans, suggesting that SPECT may be able to reveal functional alterations before the occurrence of appreciable morphological or structural damage (74). Lappalainen et al. performed perfusion SPECT and electroencephalography (EEG) on 13 patients with RTT and found that frontal hypoperfusion was consistent with frontal paroxysmal activity on EEG, and these two alterations were associated with disease progression (73). Finally, Naidu et al. performed PET studies using ^15^O-labeled water and observed a decreased blood flow in the frontal regions, similar to the observations made by Yoshikawa et al. in patients with RTT (6, 76).

Although SPECT and PET are excellent methods for measuring CBF, it is difficult to justify their use in young children who were given the radioactivity. However, recent developments in novel MRI approaches provide an excellent alternative to PET for measuring CBF. Naidu et al. used a transfer-insensitive labeling technique to measure CBF based on the concept of ASL (58), revealing greater hypoperfusion in the frontal lobe compared to other brain regions (6).

## Brain Functional Imaging

PET is helpful not only for brain blood perfusion but also for brain functional imaging. Due to its associated radiation exposure, the use of PET to assess pediatric neurodevelopmental disorders has been greatly limited. Therefore, PET has a vast unexplored potential to improve our understanding of the pathophysiology of neurodevelopmental disorders in children (77). PET biomarkers can be applied to disease diagnosis, clinical progression monitoring, and treatment response evaluation.

### Glucose Metabolism Imaging

^18^F-fluorodeoxyglucose (^18^F-FDG) PET has been introduced to evaluate human cerebral glucose metabolism and has shown that glucose utilization undergoes dramatic temporal-spatial changes. Villemagne et al. studied glucose metabolism with PET in six girls with RTT aged 3–15 years and found relatively increased glucose metabolism in the frontal cortex of younger study participants (3–8 years of age) (78). A postmortem report showed increased N-methyl-D-aspartate (NMDA) glutamate receptors in the superior frontal gyrus in young RTT subjects (79, 80). These studies showed that increased glutamate–glutamine neurotransmitter cycling at synapses accounted for the increased glucose levels in the frontal regions. A few studies have also found that glucose metabolism is relatively decreased in the visual association areas of the occipital lobe and increased in the cerebellum in RTT subjects compared to normal control subjects (81). This finding was consistent with the developmental delays observed in girls with RTT as the metabolic alterations occurred during the developmental period, particularly in children aged <1 year.

Despite these significant advancements, the relationship between CBF and glucose metabolism is still unclear. The reasons for this phenomenon might be, on one hand, the increased NMDA glutamate receptors observed in the superior frontal gyrus, and on the other hand, the observation that postsynaptic neurons respond poorly to excitatory neurotransmitters (6).

### Neurotransmitter Receptor Imaging

PET may be used to noninvasively assess gene expression either at the messenger RNA or protein expression levels using specific molecular imaging probes to quantitatively study the dynamic processes *in vivo*. Therefore, it is an urgent need to develop more specific PET imaging agents suitable for cerebral target imaging (77).

#### Nigrostriatal Function Imaging

PET imaging with the targeted agents has been applied to investigate a nigrostriatal function in patients with RTT. Using multimodal PET imaging, Henry et al. reported that in patients with RTT the mean of ^18^F-fluoro-L-dopa uptake values was reduced by 12% in the putamen and 13% in caudate nuclei compared to age-matched healthy subjects, while ^11^C-raclopride (which acts as an antagonist on D_2_ dopamine receptors (D_2_Rs)) mean uptake values were increased by 10% in the same regions (53). This divergence between dopamine reduction and D_2_Rs increase suggests that dopamine reduction decreases dopaminergic activity and thus increases compensatory D_2_R activity. These observations also suggest the existence of a presynaptic deficit of nigrostriatal activity, which could be a potential biomarker to monitor disease progression in RTT (53, 77).

Naidu et al. studied 12 adult patients with RTT using ^11^C-N-methyl-spiperone PET imaging and found low levels of postsynaptic D_2_Rs in caudate (6). Wong et al. reported decreased D_2_R density in women aged 15–30 years with RTT (82). These findings contrast with the observations of Chiron et al. who reported a markedly increased specific binding of ^123^I-iodolisuride to D_2_Rs in 11 children with RTT in the age range from 4 to 15 years (75). These studies suggest the existence of significant age-related changes in D_2_Rs—that is, patients may have higher D_2_R densities than normal subjects in the first decade of life but lower D_2_R densities as they approach adulthood. The abovementioned studies demonstrated that a steady developmental dopaminergic imbalance develops as patients age, consistent with the clinical features of increased muscle tone and rigidity seen in this disease (75, 82).

Further studies have used PET to quantify dopamine transporter (DAT) and D_2_Rs. In human studies, researchers found a significantly reduced DAT in the caudate nuclei of women with RTT compared to control subjects and reduced D_2_R numbers in the striata of women with RTT (6, 82). Wong et al. demonstrated a significant reduction in D_2_R density in the striatum of women with RTT compared to controls, but no significant differences in DAT density were observed when partial volume corrections were applied. In animal studies, Wong et al. also found a significant decrease in D_2_R and DAT density with the SRTM analysis in *MECP2*-null mice and HET mice compared to wild-type mice. The above results confirm that reductions in D_2_R are more likely to explain ambulation impairments and progressive rigidity than alterations in DAT (82). Together, these PET findings add to our understanding of the pathophysiology of RTT and provide the avenues of research that could lead to the discovery of valid biomarkers (77).

#### Histone Deacetylase Function Imaging

Histone deacetylase 6 (HDAC6) is a histone deacetylase paralogue. Its function and dysregulation correlate with the etiology of neurodevelopmental disorders, including RTT. In RTT mouse models, the upregulation of the HDAC (1 and 2) repressor complex has been found to be implicated in disease etiology. A PET probe of HDAC6 has an excellent potential to provide new insights into brain functional molecular mechanisms and facilitate the identification of therapeutic targets. A highly brain-penetrant HDAC6 inhibitor, bavarostat, exhibits excellent HDAC6 selectivity. Bavarostat radiolabeling with ^18^F by deoxyfluorination has been demonstrated to be suitable for mapping HDAC6 in the living brain in rodent and nonhuman primate models. Meanwhile, it has been shown to exhibit a high uptake in the brain, providing a key tool to study HDAC6 in the living human brain (83). Therefore, ^18^F-bavarostat may show great promise as a radiotracer in *MECP2*-defect mouse models and patients with RTT.

#### Gamma-Aminobutyric Acid Receptor Imaging

Previous studies have demonstrated that GABAergic dysfunction is a critical mediator of RTT phenotypes. *MECP2* deficiency in GABAergic neurons further leads to a series of clinical symptoms in RTT, including stereotyped movements, compulsive grooming, increased sociability, impaired motor coordination, learning/memory deficits, abnormal EEG hyperexcitability, severe respiratory dysrhythmias, altered sensorimotor gating and arousal, and premature lethality (32, 84). These details are described in Supplementary Table S3. Compared to age-matched control subjects, a few studies have demonstrated abnormal densities of gamma aminobutyric acid (GABA) receptors in the postmortem brain tissue from young female individuals with RTT. Therefore, it is of great significance to study the changes of GABA receptors *in vivo* using neuroimaging.

PET/SPECT approaches with specific imaging agents binding GABA or benzodiazepine (BZ) receptors allow us to investigate their distribution *in vivo* (85). However, the PET/SPECT imaging agents currently available for human use are more likely to bind to BZ receptors than to GABA_A_ receptors. Existing PET/SPECT imaging agents, like iomazenil and flumazenil, have limited subunit selectivity, binding to GABA_A_/BZ receptors containing multiple subunits, whereas ^11^C Ro15-4513 (a GABA_A_/BZ receptor inverse agonist) has more selectivity for α1 and α5 (86).

Yamashita et al. evaluated BZ receptor binding in the brain of adult patients with RTT using ^123^I-iomazenil SPECT imaging (87) and found that BZ receptor binding was significantly decreased in the frontotemporal cortex of patients with RTT, and subsequently in the occipital and parietal cortical GM than in the brain of five healthy male volunteers. Their study was the first to demonstrate that GABA/BZ receptor-mediated neurotransmission is inhibited in adult patients with RTT (87). However, the abovementioned analyses were performed on adult neurons, and the receptor-binding potential in young patients requires further evaluation.

Researchers used ^11^C-flumazenil PET to examine GABA_A_ receptor-binding abnormalities in patients with Angelman syndrome and confirmed a significantly decreased uptake of ^11^C-flumazenil in frontal, parietal, hippocampal, and cerebellar regions compared to the effects of a patient with a *GABRB3* gene deletion (88). Lucignani et al. studied six adults with Prader–Willi syndrome and found a decreased uptake of ^11^C-flumazenil in the insula and cingulate, frontal, and temporal neocortices than in normal control subjects (89). In previous studies, GABAergic dysfunction was confirmed in patients with RTT. Theoretically, high-resolution PET GABA receptor imaging to examine GABA_A_ receptor-binding abnormalities in patients with RTT is feasible.

Proton MRS used for GABA detection can measure GABA concentrations within a voxel of interest. This approach theoretically measures the total GABA contents of the voxel (that is, the intracellular and extracellular contents and those involved in metabolism or neurotransmission). It cannot be discriminated between GABA levels in different cell types, which limit its application in addressing cell- and network-specific GABA abnormalities. The development of the MEGA-PRESS sequence can quantify GABA concentrations in the human brain *in vivo*. Meanwhile, GABA_A_/BZ receptor PET imaging may measure the changes in synaptic GABA concentrations.

In the future, the combination of GABA PET with proton MRS in the same subjects on PET–MRI platforms might be more accurate in investigating the dysfunction of synaptic vs. nonsynaptic GABA in RTT (90).

#### Magnetic Resonance Spectroscopy

*In vivo*, MRS can detect important cerebral metabolites, including N-acetyl aspartate (NAA), total choline (Cho), total creatine (Cr), and glutamate/glutamine, offering the potential to reveal regional cerebral metabolisms in RTT noninvasively. MRS has revealed decreased NAA levels in both the GM and WM (6). The identified regional metabolic abnormalities include significantly lower NAA concentrations in frontal and parietal lobes, the insular cortex, and the hippocampus in RTT, reflecting a reduced neuronal and dendritic size and decreased neuronal function (91). Studies have reported that the average Cho concentration was higher in patients with RTT possibly due to gliosis than the control group, but there were no significant differences in regional Cho and Cr concentrations. There was a higher Cho/NAA ratio in the frontal and parietal GM/WM, insular GM, and hippocampus and a lower NAA/Cr ratio in the frontal cortical GM, parietal and temporal WM, insula, and putamen of RTT subjects compared to controls (92). Increased glutamate in MRS studies suggests the presence of increased glutamate–glutamine neurotransmitter cycling at the synapses in RTT, consistent with the increased glucose levels recorded in the frontal regions in PET studies and the increased glutamate/N-methyl-D-aspartate receptors identified in postmortem studies (93, 94).

Magnetic resonance spectroscopy detected the abovementioned cerebral metabolites and has revealed the distribution of other metabolites. In animal studies, a low level of Myo-inositol measured by MRS was a characteristic of the mouse model of RTT (46). One ^31^P MRS study revealed a compelling reduction in ATP and phosphocreatine (PCr) in *MECP2*-null mice that may account for the mitochondrial pathogenesis and reflect significant impairments in brain energy metabolism (46). Researchers have detected important brain anatomical and metabolic differences between C57Bl/6 and *MECP2*-/y mice using a multimodal MRI/MRS approach (46). Animal studies can lay the foundation for applying multimodal imaging in humans with RTT.

## Discussion And Future Directions

While there is still no cure for RTT, the fundamental research discoveries achieved over the past few decades have enabled to set the basis for the development of new potential therapies. Therapeutic approaches for RTT are divided into the following three categories: symptom treatment, pharmacological modulators of downstream *MECP2* targets, and genetic interventions (95). Therefore, it is necessary to search for neurofunctional markers to track drug safety and treatment response. A longitudinal brain MRI study of RTT has shown that MRI may reveal the efficacy of treatment interventions on the neuroanatomy, particularly across the critical neural networks that govern classical RTT symptoms (96).

Furthermore, early diagnosis and concerted rehabilitation efforts will be essential for improving the efficacy of therapies for RTT. Meanwhile, as RTT is characterized by complex clinical symptoms progressing through the different stages over time and varying from one individual to another, clinicians need novel measures that can reflect multilevel changes at several levels (95). Being at the interception between etiology, clinical diagnosis, and treatment, neuroimaging applications to RTT need to be further developed. In this review, we have summarized a few imaging literature studies on RTT with *MECP2* mutations and compared various imaging modalities to clarify their strengths and weaknesses (Table 2). As only a few studies have been conducted in this series of patients, this field of research should still be considered in its early stages. We, therefore, believe that there is still ample space for further neuroimaging studies on RTT, taking into account the following research priorities.

**Table 2:** Strengths and weaknesses of various imaging modalities in RTT with *MECP2* mutation^*^.

**Imaging modalities**	**Strengths**	**Weaknesses**
**Morphologic MRI**		
Structural analysis	High spatial resolution and contrast, great gray/white matter delineation	Poor contrast in younger population, especially children
Quantitative analysis	Find changes in surface or volume of multiple brain regions	under 1 year old, so disadvantageous for whole-brain analysis
**Diffusion MRI**		
DTI	Accurately characterize brain microstructure *in vivo*, high sensitivity	Non-specific, can't adequately model biological system
NODDI	More precise delineate microstructure, high sensitivity and specificity	High requirements on machine, sequence and image capture
Tractography	More precise delineation of specific fiber pathway	High requirements on image captures and post-processing
**CBP imaging**		
SPECT/PET	Excellent method for measuring CBF, semi-quantitative analysis	Radioactivity limits its use in young children, low resolution
ASL	No radioactivity, noninvasive, repeatability, quantitative	High image require, whole-brain coverage scan takes long time
**Metabolism imaging**		
SPECT/PET	High sensitivity and specificity, target imaging, quantitative	Radioactivity, specific imaging agents are difficult to develop
MRS	No radioactivity, noninvasive, high specificity, quantitative	Difficult to develop imaging sequences for specific substances

* *ASL, arterial spin labeling; CBF, cerebral blood flow; CBP, cerebral blood perfusion; DTI, diffusion tensor imaging; MECP2, methyl-CpG binding protein gene 2; MRI, magnetic resonance imaging; MRS, magnetic resonance spectroscopy; NODDI, neurite orientation dispersion and density imaging; PET, positron emission tomography; RTT, Rett syndrome; SPECT, single positron emission CT*.

**Figure 1:**
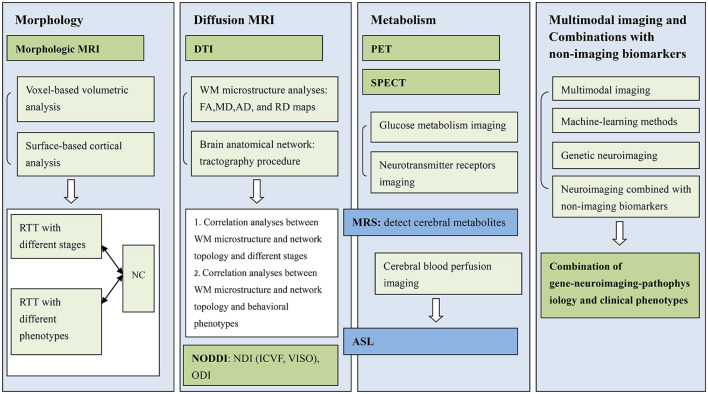
Future directions of multimodal neuroimaging in Rett syndrome (RTT) with *MECP2* mutation.

First, we must improve research on MRI morphologic imaging of patients with RTT by comparing the identified characteristics to those of the normal development population and summarizing the imaging characteristics of abnormal brain morphologic development in patients with RTT with different disease stages and phenotypes (97). Second, NODDI technology will be used to explore the imaging characteristics of brain microstructural changes in patients with RTT at different stages and phenotypes. It is expected that NODDI technology will play a significant role in the early diagnosis of this disease and the evaluation of therapeutic efficacy. Third, basic studies have discovered that *MECP2* gene mutations lead to abnormalities in many downstream neurons and related nerve signaling pathways, but the detailed mechanism is still not fully clarified, and it is necessary to develop specific neuroimaging methods/sequences or multimodal imaging to dynamically observe the abovementioned changes in the brain *in vivo*. Pharmacological modulators of downstream *MECP2* targets are being developed, and neuroimaging will play an essential role in future patient-specific drug selection and drug efficacy evaluation. Fourth, multiple MRI modalities (multimodal MRI/MRS approach, fusion imaging with ASL and dMRI, or fusion imaging with MRI and PET) and various learning algorithms, like the combination of NODDI and surface-based analyses, have been designed to provide personalized data. Machine-learning methods, such as deep learning-based segmentation of brain tissues from dMRI, have been proposed and achieved a high degree of accuracy (98), which will further apply to RTT. Lastly, exploring combinations with nonimaging biomarkers and further identifying those biomarkers' longitudinal trajectories and orders will point to the most potential combinations (99). Calabrese et al. used diffusion tensor magnetic resonance histology to track microstructural changes in the rat brain throughout normal postnatal neurodevelopment and then correlated these changes with the changes in the cytoarchitecture. They also provided a comprehensive database of image sets as a foundation for future studies (2). Consequently, a combination of gene-neuroimaging-pathophysiology and clinical phenotype analyses can effectively characterize disease states in the RTT population (Figure 1).

## Conclusion

In this review, we have attempted to summarize the findings from the conducted MRI to PET studies over the past few decades on RTT with *MECP2* mutations. MRI morphologic imaging is particularly sensitive to brain structural abnormalities in patients with RTT, while dMRI provides valuable information on brain microstructural changes and fMRI enables elucidating the underlying dysfunctional mechanisms. Finally, MRS and PET modalities play a crucial role in the exploration of metabolic alterations in this complex neurodevelopmental disease. We emphasize that the field has not flourished in the area of pediatric disorders compared to adult neurodegenerative disorders. There also remains an enormous opportunity to improve our understanding of RTT through molecular imaging with MRI and PET technology. These advances will be of great significance for the clinical diagnosis of RTT and the formulation of individualized treatment plans.

## Author Contributions

YK: guarantor of integrity of the entire study, study concepts and design, and manuscript preparation. NC, ND, Q-bL, and X-fJ: literature research. G-qZ and Z-hY: manuscript editing. All authors contributed to the article and approved the submitted version.

## Funding

This work was supported by the PhD Research Foundation of the Affiliated Hospital of Jining Medical University (Grant No. 2016-BS-016).

## Conflict of Interest

The authors declare that the research was conducted in the absence of any commercial or financial relationships that could be construed as a potential conflict of interest.

## Publisher's Note

All claims expressed in this article are solely those of the authors and do not necessarily represent those of their affiliated organizations, or those of the publisher, the editors and the reviewers. Any product that may be evaluated in this article, or claim that may be made by its manufacturer, is not guaranteed or endorsed by the publisher.

## Supplementary Material

Supplementary Table S1 Imaging studies of patients in Rett syndrome with *MECP2* mutation.

Supplementary Table S2 Animal studies of imaging in Rett syndrome with *MECP2* mutation.

Supplementary Table S3 Pathophysiological mechanism, clinical phenotypes, and therapeutic targets in different neurons with *MECP2* deletions.
