# Supplementary material for: Multimodal Neuroimaging in Rett Syndrome With MECP2 Mutation
Source: Front Neurol. 2022 Feb 23;13:838206. doi: 10.3389/fneur.2022.838206 (PMC8904872; doi:10.3389/fneur.2022.838206)
Supplement: Supplementary Table S1 — Imaging studies of patients in Rett syndrome with MECP2 mutation. [file Table_1.pdf]

Supplementary Table S1. Imaging studies of patients in Rett syndrome with *MECP2* mutation

Imaging modality	Imaging phenotype	Samples size (mean age in years)	Primary results	Reference
**MRI**	Dimensions of cerebral, cerebellum, and brainstem structures	13 RTT (12.0); 10 HC (15.4)	↓ Global hypoplasia of brain (cerebral, cerebellum, corpus callosum and brainstem)<br>↓ Progressive cerebellar atrophy increasing with age	Murakami et al. 1992 (52)
	TBV, cortical GM and WM<br>Subcortical gray nuclei<br>CSF volumes	11 RTT (10.1); 15 HC (11.2)	↓ Volumes in cerebrum, basal ganglia, and brainstem<br>↓ Loss of GM in comparison to WM, with largest decrease in frontal regions and CN and midbrain volume	Reiss et al. 1993 (43)
	TBV, cortical GM and WM,<br>Subcortical GM, CSF and posterior fossa volumes	20 RTT (9.8); 20 HC (9.0)	↓ GM volume most pronounced in prefrontal, posterior-frontal, and anterior-temporal regions<br>↓ WM volume uniformly throughout brain<br>↓ CN volume<br>No differences in midbrain volumes	Subramaniam et al. 1997 (44)
	Quantitative shape analysis	8 RTT (5.3); 10 HC (7.0)	↓ Whole brain hemisphere<br>↓ Both right and left caudate	Manuel et al. 1991 (51)
	Absolute and relative changes in GM and WM volumes	22 RTT (8.6) ((12 more severe (8.8); 10 less severe (8.3)); 25 HC (8.9)	↓ TBV<br>↓ Relative parietal lobe GM volume, particularly dorsal<br>↓ Cortical WM volume<br>↓ Anterior frontal lobe volumes in more	Carter et al. 2008 (50)

			severely affected subjects	
	Surface- and voxel-based brain morphological measurements including cortical thickness and cortical gyrification, global/regional GM and WM volumes	7 RTT (5.2); 16 HC (gender- and age-matched)	↓ Decreased total volumes of the cerebellum.<br>No differences in global cerebral cortical surface areas, global/regional cortical thicknesses, the degree of global gyrification, and global/regional gray and white matter volumes	Shiohama et al. 2019 (54)
	Volumetric measurements of basal ganglia	9 RTT (18.4); 9 HC (20-29 years)	↓ Significant reduction in the size of the caudate heads and thalami	Dunn et al. 2002 (53)
**DTI**	FA	32 RTT (5.5); 37 HC (6.1)	↓ FA in genu and splenium of CC and external capsule, and regions of cingulate, internal capsule, posterior thalamic radiation, and frontal WM.<br>No differences in visual pathways<br>↓ FA in superior longitudinal fasciculus in patients who were nonverbal or speaking only single words	Mahmood et al. 2010 (68)
	FA	9 RTT; 13 HC (gender- and age-matched)	↓ FA in the left peripheral white matter areas (the middle temporal, middle occipital, pre-cuneus, and the post-central white matter)<br>↓ FA in left major white matter tracts (the superior longitudinal fasciculus, sagittal stratum, corpus callosum)	Oishi et al. 2013 (48)

			↓ FA in the bilateral cingulum	
**MRS**	NAA, Glu/Gln, Cho, Cre, and GABA	9 RTT (9.9)	↓ NAA decreased with increasing age<br>↓ Glu/Gln decreased<br>↓ Cho, Cre slightly decreased<br>↑ GABA increased	Hanefeld et al. 1995 (94)
	Glu, Cr, and NAA	6 RTT (4.5-6 years); 4 HC (5.5-13 years)	↓ NAA level reduced<br>↑ Cr/NAA ratio was elevated in WM, and normal in GM<br>↑ Glu/NAA ratio was elevated in GM, and normal in WM	Pan et al. 1999 (93)
	NAA, Cr, Cho, and mI peaks were quantitatively evaluated NAA/Cr, NAA/Cho, and Cho/Cr, mI/Cr ratios were calculated	7 RTT (7.7); 5 HC (6.4)	↓ NAA level reduced<br>↓ Decreases in NAA/Cr, and NAA/Cho radios.<br>No differences in Cho/CR ratios and mI/Cr ratios.	Gokcay et al. 2002 (92)
**SPECT**	Brain blood flow detected with ^{133}Xe SPECT	7 RTT (10.1); 9 HC (13.6)	↓ Global CBF<br>Hypoperfusion foci were located mainly in the prefrontal and temporoparietal areas	Nielsen et al. 1990 (72)
	Brain blood flow detected with ^{99m}Tc-ECD SPECT	12 RTT (11.4); 9 HC (7.3)	↓ Global reduction in cerebral perfusion, especially in the frontal and fronto-parietal regions bilaterally<br>↓ Brain perfusion abnormalities were more often in stage IV than the stage III<br>No significant right-to-left asymmetry was found in any ROIs of the cortex	Burroni et al. 1997 (74)

	rCBF detected with ^{99m}Tc-HMPAO SPECT	13 RTT (8.4); 9 HC (-)	↓ Frontal hypoperfusion was the most frequent abnormality<br>↓ Parietal and temporal hypoperfusion was detected in some patients<br>No hypoperfusion in occipital lobes	Lappalainen et al. 1997 (73)
	rCBF detected with ^{133}Xe SPECT	11 RTT (7.9); 8 HC (8.1)	No significantly difference in young RTT patients	Chiron et al. 1993(75)
	^{123}I-Iodolisuride detected striatal D_2 receptors	11 RTT (7.9); 8 HC (8.1)	↑ Increased ^{123}I-Iodolisuride uptake in the striatum	Chiron et al. 1993(75)
	Evaluate BZD receptor binding in adult females with stage IV RTT by using ^{123}I- iomazenil	3 RTT (34.3); 5 HC (24-61 years)	↓ A decreased in BZD receptor binding in the cortex of adult patients with RTT<br>↓ The BP for the BZD receptor was significantly decreased in the fronto-temporal cortex of the RTT patients	Yamashita et al. 1998 (87)
**PET**	CBF, $CMRO_2$	6 RTT (8.7); 3 HC (6.3)	↓ CBF reduced in the frontal cortex and the temporal cortex<br>↓ $CMRO_2$ reduced in the frontal cortex and the temporal cortex	Yoshikawa et al. 1991 (76)
	CBF, $CMRO_2$ by using ^{15}O labeled water	4 adult RTT; age-matched HC	↓ CBF in the frontal regions	Naidu et al. 2001 (6)
	Uptake of ^{18}F-FDG	6 RTT (9); 18 HC (age-matched)	↑ Significant increase in relative glucose utilization was observed in the frontal areas in the younger group<br>↑ Increase in cerebellar glucose metabolism in both age group<br>↓ The lower relative glucose metabolism	Villemagne et al. 2002 (78)

			detected in the occipital visual association areas in both age group	
	Uptake of ^{18}F-FDG	RTT compared with HC	↓ Decrease in visual association areas of the occipital lobe<br>↑ Raised in the cerebellum	Naidu et al. 1992 (81)
	Uptake of ^{18}F-6-fluorodopa and ^{11}C-raclopride	9 RTT (18.4); 9 HC (20-29 years)	↓ Fluorodopa was reduced in caudate and putamen<br>↑ Dopamine D_2 receptor binding was increased in caudate and putamen	Dunn et al. 2002 (53)
	Uptake of ^{11}C-N-methyl-spiperone	12 RTT (15-39); HC (age-matched)	Low normal levels of post-synaptic D_2Rs in caudate	Naidu et al. 2001 (6)
	3-N-^{11}C-methylspiperone uptake detect D_2R	10 RTT (20.7); 16 HC (age-matched)	↓ D_2R was significantly lower in women with RTT with no significant age-related changes	Wong et al. 2018 (82)
	^{11}C-WIN35,428 uptake detect DAT	9 RTT (20.8); 8 HC (age-matched)	↓ DAT density was lower in the caudate in women with RTT	Wong et al. 2018 (82)

^{18}F-FDG, fluorine 18-fluorodeoxyglucose; PET, positron emission tomography; BZD, benzodiazepine; CBF, cerebral blood flow; CC, corpus callosum; Cho, choline; CMRO, cerebral metabolic rate of oxygen; CN, caudate nudeus; Cr, creatine; CSF, cerebrospinal fluid; D_2R, D_2 dopamine receptor; DAT, dopamine transporter; DTI, diffusion tensor imaging; FA, fractional anisotropy; GM, gray matter; HC, health control; *MECP2*, methyl-CpG binding protein gene 2; MRI, magnetic resonance imaging; MRS, magnetic resonance spectroscopy; NAA, N-acetyl aspartate; ROI, regions of interest; RTT, Rett syndrome; SPECT, single positron emission computed tomography; TBV, total brain value; WM, white matter.
