# Supplementary material for: Multimodal Neuroimaging in Rett Syndrome With MECP2 Mutation
Source: Front Neurol. 2022 Feb 23;13:838206. doi: 10.3389/fneur.2022.838206 (PMC8904872; doi:10.3389/fneur.2022.838206)
Supplement: Supplementary Table S2 — Animal studies of imaging in Rett syndrome with MECP2 mutation. [file Table_2.pdf]

Supplementary Table S2. Animal studies of imaging in Rett syndrome with *MECP2* mutation

Imaging modality	Imaging phenotype	Animal model	Primary results	Reference
**MRI**	Volume measurement	Strain B6.129P2(C)-<br>*MECP2*$^{tm1-1Bird}$<br>*MECP2 -/y* male mice	↓ A global reduction in brain size<br>↓ A reduction in cerebellum size<br>↓ Significantly thinner in some specific structures, especially the motor cortex and the corpus callosum	Saywell et al. 2006 (46)
	Volume measurement<br>Segmentation of the mouse cerebellum in MRI cerebellar atlas	Homozygous females (*MECP2* 308 −/−), heterozygous females (*MECP2* 308 −/+), hemizygous males (*MECP2* 308 −/Y), and non-littermate WT from a C57BL/6 background.	The *MECP2* mutant mice had cerebellar volume changes that increased in scope depending on the genotype: hemizygous males to homozygous females	Steadman et al. 2014 (49)
	Voxelwise and regional-based analysis within segmented anatomical regions to determine the location, direction, and magnitude of the neuroanatomical differences	Male and female mice from the *MECP2*^{tm1Hzo}, *MECP2*$^{tm1.1Bird/J}$, and *MECP2*$^{tm2Bird/J}$ mouse lines	↓ Regardless of mutation type, regional volumes of the frontal, cingulate, sensory, motor cortices, the striatum, thalamus, and white matter tracts were smaller in mutant mice relative to their WT controls<br>Regions of the cerebellum were differentially affected by the type of mutation:<br>↑ An increase in volume in the mutant *MECP2*^{tm1Hzo} brain relative to controls<br>↓ A decrease volume in the *MECP2*$^{tm1.1Bird/J}$ and *MECP2*$^{tm2Bird/J}$ lines	Allemang-Grand et al. 2017 (47)

**DTI**	FA, MD, AD, and RD were calculated<br>Network topological organizations	Female RTT monkeys were generated using TALENs-based mutagenesis technique	↓ Decreased FA and increased RD values in WM tracts of bilateral posterior temporal, parietal, ventroposterior frontal, and right medial occipital lobes, as well as subcortical areas including striatum and thalamus<br>↓ Decreased FA and increased RD values of bilateral cingulum and corpus callosum<br>Protracted early WM myelinization	Wang et al. 2021 (70)
**PET**	^{11}C-raclopride for D_2R imaging<br>^{11}C-MP for DAT analysis	*MECP2*-null mice, HET mice, WT mice	↓ Significantly reduced in HET mice and in *MECP2*-null mice compared to WT mice<br>DAT varied by the type of analysis model used:<br>↓ The SRTM method showed a significant decrease in the BD_{ND} in *MECP2*-null mice compared to WT<br>The LOGAN analysis model found significant age-related changes in BD_{ND} in WT mice that were not observed in *MECP2*-deficient mice	Wong et al. 2018 (82)
**MRS**	^{1}H MRS<br><br><br>^{31}P MRS	Strain B6.129P2(C)-*MECP2*$^{tm1-1Bird}$<br>*MECP2 -/y* male mice	↓ The low level of NAA, myo-inositol, and glutamine plus glutamate in *MECP2 -/y* mice ↑ Increased choline levels<br>↓ Reduction in ATP and PCr	Saywell et al. 2006 (46)

^{11}C-MP, ^{11}C-methylphenidate; AD, axial diffusivity; BP, binding potential; Cho, choline; Cr, creatine; D_2R, D_2 dopamine receptor; DAT, dopamine transporter; DTI, diffusion tensor imaging; FA, fractional anisotropy; *fin*, intraneurite volume fraction; GM, gray matter; HET, heterozygous; *irfrac*, isotropic

restricted volume fraction; *fiso*, isotropic fraction; MD, mean diffusivity; *MECP2*, methyl-CpG binding protein gene 2; MRI, magnetic resonance imaging; MRS, magnetic resonance spectroscopy; NAA, N-acetyl aspartate; ODI, orientation–dispersion index; PCr, phosphocreatine; PET, positron emission tomography; RD, radial diffusivity; RTT, Rett syndrome; WM, white matter; WT, wild type.
