# Supplementary material for: Multimodal Neuroimaging in Rett Syndrome With MECP2 Mutation
Source: Front Neurol. 2022 Feb 23;13:838206. doi: 10.3389/fneur.2022.838206 (PMC8904872; doi:10.3389/fneur.2022.838206)
Supplement: Supplementary Table S3 — Pathophysiological mechanism, clinical phenotypes, and therapeutic targets in different neurons with MECP2 deletions. [file Table_3.pdf]

Supplementary Table S3. Pathophysiological mechanism, clinical phenotypes, and therapeutic targets in different neurons with *MECP2* deletions

Neuron types	Pathophysiological mechanism	Clinical phenotypes	Therapeutic targets
**Dopaminergic neurons**	Affect dopaminergic nervous system	Motor disorder: stereotyped hand-wringing, progressive rigidity, dyskinesia, and dystonia	Dopaminergic agents: levodopa and benserazide
**Cholinergic neurons**<br>The basal forebrain-hippocampus<br>The striatum	 Affect cholinergic neural circuit<br>Up-regulation of α2-GABA receptor	Anxiety/depression-related behavioral changes, abnormal social skills, susceptibility to epilepsy, and loss of fear memory	Cholinergic Drugs: Choline
**GABAergic neurons**<br>Brain stem / basal forebrain<br>The hippocampus	GABAergic signaling dysfunction	Repetitive behaviors, impaired motor coordination, increased social interest, and altered sensorimotor gating and arousal	GABAergic modulators:<br>$GABA_A$ antagonist<br>GABA reuptake inhibitors
**Forebrain excitatory neurons**	Down-regulation GABA receptor	Motor disorder, increased anxiety-like behaviors and impaired fear conditioning and social behavior	GABAergic modulators
**Glutamatergic neurons**<br>The frontal cortices<br>The hippocampus	Glutamatergic signaling dysfunction leads to repetitive glutamate transients	The motor performance, clasping behavior and fear-conditioning defects, sleep dysfunction, susceptibility to epilepsy	Glutamatergic modulators:<br>NMDA receptor antagonist<br>mGlu5 modulator
**BDNF expression neurons**<br>The cerebral cortex, hippocampus and cerebellum	BDNF-TrkB signaling dysfunction:<br>Decrease in BDNF and TrkB receptor protein levels	Motor deficits, breathing dysfunction, heart rate disorder, neural morphology abnormality, life span influence	Growth factor therapy<br>BDNF mimetics or boosters
**HDAC expression neurons**	HDAC overexpression and decrease in acetylated tubulin levels<br>Affect BDNF signaling	Motor deficits, breathing dysfunction, heart rate disorder, neural morphology abnormality, life span influence	HDAC inhibitors
**Serotoninergic neurons**	Down-regulation serotonin receptor	Increased aggression, irregular breathing and apnoea	Serotonin receptor agonist<br>Dopamine D_2 partial agonist
**Catecholaminergic neurons**<br>Neurons in the locus coeruleus	Norepinephrine system dysfunction	Impairment of cognitive and attention, anxiety, psychosis, motor disorder, breathing	Adrenergic receptor agonist

The brain stem		dysfunction, and social abnormalities	
***Sim1*-expressing neurons in the hypothalamus**	Disruptes MC4R signaling pathway<br>Decreased expression of *Crh* and BDNF in PVN	Alterations in feeding behavior, aggression and stress response	Adrenergic drugs<br>BDNF mimetics or boosters
**Somatosensory neurons in the barrel cortex**	Impaired E/I balance	Leads to anxiety, social interaction abnormalities, and tactile sensory and motor problems	Antidepressants

BDNF, brain-derived neurotrophic factor; *Crh*, Corticotropin-Releasing Hormone; E/I, excitation/inhibition; GABA, γ-aminobutyric acid; HDAC, histone deacetylase; *MECP2*, methyl-CpG binding protein gene 2; mGlu5, metabotropic glutamate 5 receptor; NMDA, *N*-methyl-D-aspartate; PVN, paraventricular nucleus; TrkB, tyrosine receptor kinase B.
